# High-Resolution Modeling of Uplift Landscapes can Inform Micrositing of Wind Turbines for Soaring Raptors

**DOI:** 10.1007/s00267-020-01318-0

**Published:** 2020-06-24

**Authors:** Frank Hanssen, Roel May, Torgeir Nygård

**Affiliations:** Norwegian Institute for Nature Research, P.O. 5685 Torgarden, 7485 Trondheim, Norway

**Keywords:** White-tailed eagle, Norway, Thermal and orographic updraf, GIS, Remote sensing

## Abstract

Collision risk of soaring birds is partly associated with updrafts to which they are attracted. To identify the risk-enhancing landscape features, a micrositing tool was developed to model orographic and thermal updraft velocities from high-resolution remote sensing data. The tool was applied to the island of Hitra, and validated using GPS-tracked white-tailed eagles (*Haliaeetus albicilla*). Resource selection functions predicted that eagles preferred ridges with high orographic uplift, especially at flight altitudes within the rotor-swept zone (40–110 m). Flight activity was negatively associated with the widely distributed areas with high thermal uplift at lower flight altitudes (<110 m). Both the existing wind-power plant and planned extension are placed at locations rendering maximum orographic updraft velocities around the minimum sink rate for white-tailed eagles (0.75 m/s) but slightly higher thermal updraft velocities. The tool can contribute to improve micrositing of wind turbines to reduce the environmental impacts, especially for soaring raptors.

## Introduction

While the development of wind energy contributes to reducing greenhouse gas emissions, this may simultaneously negatively affect wildlife, particularly birds and bats. Internationally, there is a particular concern about birds colliding with wind turbines (Langston 2013; Marques et al. 2014; Schuster et al. 2015). Soaring bird species, such as raptors, are known to be specifically vulnerable for collision with wind turbines (Ferrer et al. 2012; Wang et al. 2015). Collision risk of soaring raptors is associated with species-specific flight behavior, topographical features, and seasonal abundances; however, the exact mechanisms of collision susceptibility remain unclear (Barrios and Rodriguez 2004; Dahl et al. 2013; de Lucas et al. 2008). At wind-power plants, that have already incurred multiple raptor mortalities, these collisions have often clustered around a limited number of turbines (Barrios and Rodriguez 2004, 2007; Ralston Paton et al. 2017). Effective mitigation actions hereby require prediction of the expected area utilization of present birdlife for the identification of sites with higher expected collision risk.

Following the rationale of the mitigation hierarchy, mitigation measures implemented early in the development process should be prioritized (May 2017). Promising approaches to facilitate “bird-friendly” micrositing of turbines during the preconstruction design, and detailed planning phase may support locating wind turbines at sites that pose minimal risk to birds (Jenkins et al. 2015; May 2017). Several studies have attempted to predict locations with increased collision risk for raptors using resource selection functions based on telemetry data (Miller et al. 2014) or simulated horizontal wind flows across the topography in an wind tunnel (de Lucas et al. 2012). However, the first approach requires detailed knowledge on movement patterns in species at risk, while the second approach requires access to a boundary layer wind tunnel to be able to predict potential risk at a planned wind energy site. At the early planning phase, such data sources or experimental infrastructure are however most likely not available.

Many bird species (especially raptors) are attracted to vertical air currents or updrafts, which allows for energy-saving soaring flight (Harel et al. 2016a; Hedenström and Alerstam 1995; Shamoun-Baranes et al. 2003, 2016). Updrafts induced by the landscape occur when solar heating of land cover types with specific spectral reflectance characteristics creates vertical air fluxes (thermal updrafts), and when horizontal wind is forced upwards by elevated topography (orographic updrafts) (Bohrer et al. 2012). The birds’ ability to exploit these updrafts (their uplift capacity) is species-specific (Mellone et al. 2012; Shamoun-Baranes et al. 2003). Updraft modeling may therefore provide a proxy for identifying sites with potentially increased risk of collision for soaring birds.

Several attempts have been made to estimate the location and intensity of such updrafts at different spatial resolutions utilizing GIS, remote sensing, weather forecast models, and computational fluid dynamic models (Bohrer et al. 2012; Harel et al. 2016b; Shamoun-Baranes et al. 2016; Shannon et al. 2003; Shepard et al. 2016; Treep et al. 2016). Estimating thermal updrafts is very complex due to the chaotic nature of turbulence governing the atmosphere (Reddy et al. 2016). Bohrer et al. (2012) estimated thermal updraft velocity from the North American Regional Reanalysis (NARR) model-observation hybrid dataset (32 × 32 km). Shannon et al. (2003) and Harel et al. (2016b) refer to the estimation of thermals based on weather forecast models such as the European Center for Medium Range Weather Forecast model (ECMWF, 12.5 × 12.5 km).

With the exception of Treep et al. (2016) and Shepard et al. (2016), the above-mentioned attempts were based on spatial datasets with coarse resolutions (>1 km). Treep et al. (2016) used a re-analyzed ECMWF model with a spatial resolution of 300 m and a digital terrain model (DTM) with a spatial resolution of 90 m. Shepard et al. (2016) used a LIDAR-based DTM with a spatial resolution of 2 m and a computational fluid dynamic (CFD) model with a spatial resolution of 1 m. Still, to enable applying these models for siting of wind turbines, the model should be spatially explicit with 1) a relative fine spatial resolution and 2) have the capability of implementing cost-effectively in a preconstruction situation. Weather models (Treep et al. 2016) represent interpolations from meteorological ground stations at coarser spatial resolutions, and therefore do not fully comply to the first requirement. LIDAR-based models (Shepard et al. 2016) will depend upon high-cost laser-scanning, and therefore hamper the second requirement.

To support bird-friendly micrositing of turbines, locations with terrain characteristics that attract soaring birds and thereby enhance the risk of collision need to be identified—and consequently avoided—at fine-scale spatial resolutions without requiring collecting site-specific data (May 2017). Scacco et al. (2019) found that static features of the landscape proved to be highly effective in identifying areas suitable for uplifts. Publicly available Landsat 8 imagery enables the quantification of reflected radiation from the surface at a relatively fine spatial resolution of 100 m. Augmentation with high-resolution DTMs (10 m) allows the fine-scale assessment of risk for soaring raptors for any planned wind energy project. The main objective of this study was therefore to identify the risk-enhancing landscape features by modeling orographic and thermal updraft velocities at such a fine spatial resolution. The updraft calculations were previously validated in Tarifa on the Spanish side of the Strait of Gibraltar (Santos et al. 2017). Here, the methodology was applied and validated in a totally different habitat and environmental conditions, and specifically its merit for micrositing of wind turbines was evaluated. The updraft landscape on the island of Hitra, Norway, was modeled using a combination of Landsat 8 Thermal Band 10 and the Norwegian DTM10 elevation model, including a validation using GPS-tracked white-tailed eagles, *Haliaeetus albicilla*. The hypothesis was that white-tailed eagles selected locations with increased orographic and thermal updraft velocities that enable soaring flight; thereby enhancing the risk of collision (i.e., through increased exposure). At these northern latitudes selection for sites with thermal uplift were expected to be significantly lower than for sites with orographic uplift, and where white-tailed eagles would rely more on the relatively stronger orographic updrafts in their soaring flight preferences. It was also hypothesized that preference for updraft sites was highest at lower flight altitudes as a means to gain altitude for the eagles (c.f. Duerr et al. 2012). Finally, a preliminary evaluation was performed on the effect of maximum updraft velocities on the collision rate at wind turbines on the island. If these expectations hold, maximum orographic and thermal updraft velocities provide the cost-effective proxies for risky locations for soaring birds. Following a precautionary approach, avoiding such locations will be important information for bird-friendly micrositing of wind turbines.

## Methodology

### Study Site

**Fig. 1:**
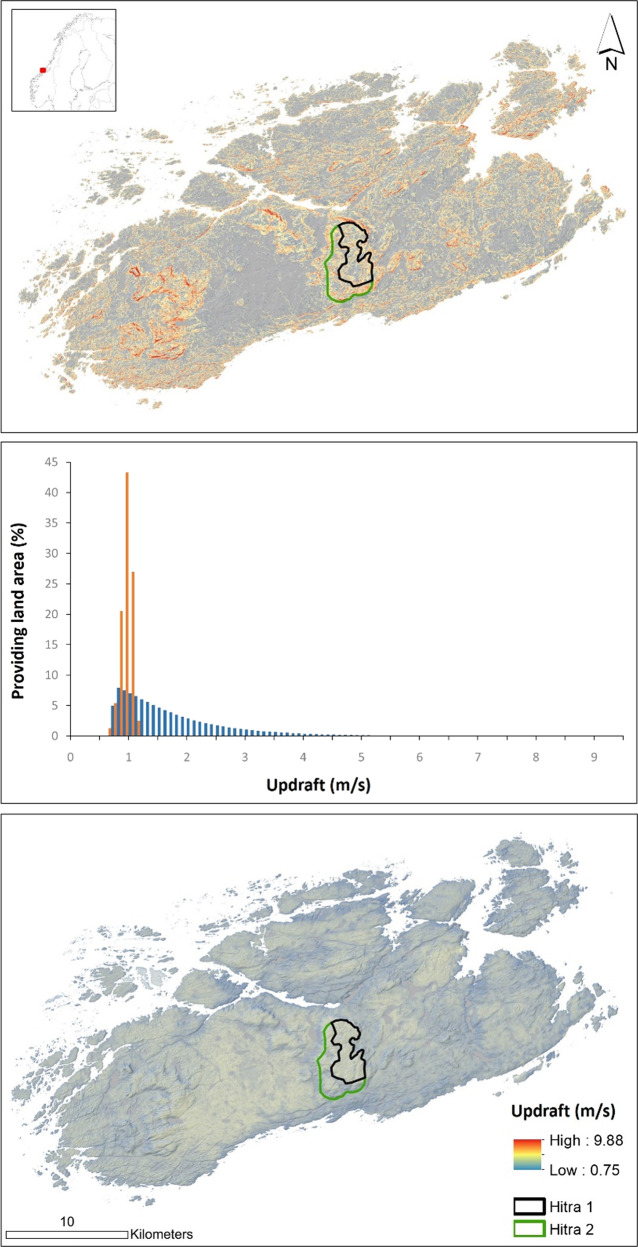
Estimated maximum orographic (top panel) and thermal (lower panel) updrafts velocities (m/s) across seasons above the minimum required sink rate required by white-tailed eagles (0.75 m/s) to be able to glide on upward moving air currents on the island of Hitra, Norway. The middle panel shows proportion of land area providing updrafts (in % of total for each updraft category separately) across updraft velocities ranging from 0.75 to 9.88 m/s for orographic (red bars) and thermal updrafts (blue bars). The existing wind-power plant constructed in 2004 and planned extension in 2019 are respectively outlined in black and green

This study was implemented on the Hitra Island (63.60° N, 8.65° E) in Trøndelag County, Norway (Fig. 1). Hitra has a typical coastal climate with relatively temperate springs/summers and mild autumns/winters dominated by strong winds and heavy rainfalls. With its land area of 680.4 km^2^, Hitra and its surrounding archipelago provides an important habitat for white-tailed eagles (Dahl et al. 2012; May et al. 2013). The terrain on Hitra is relatively rugged with 16 mountain peaks (the highest peak being 345 m above sea level). The land cover on Hitra is comprised of lakes and rivers (5%), bogs (16%), forests (29%), farmland (2%), and open areas and settlements (48%). At Eldsfjellet, a mountain area in the central part of Hitra, a wind-power plant was constructed in 2004 consisting of 24 turbines (55 MW installed capacity). In 2019, this wind-power plant will be expanded with another 26 turbines (additional 94 MW installed capacity).

### Modeling the Updraft Landscape

#### Estimation of thermal updrafts

The thermal updraft velocity was estimated at a 100 × 100 m spatial resolution using a combination of Landsat 8 imagery, climate variables and atmospheric constants. The thermal updraft velocity was estimated using the standard atmospheric scaling coefficient called the “free connectivity scaling velocity” or the Deardorrf velocity (w^*^) according to Bohrer et al. (2012):

$$w^ \ast = \left[ {\frac{{g \cdot z \cdot H}}{\theta }} \right]^{\frac{1}{3}},$$

with *g* as the gravitational acceleration (set to 9.8 m/s^2^), *z* as the flight altitude in the rotor swept zone (set to 80 m a.g.l., assumed to be within the atmospheric boundary layer), *H* as the surface sensible heat flux measured in W/m^2^ and *θ* as the potential temperature measured in Kelvin. The surface sensible heat flux (*H*) describes how thermal energy is transferred from the ground surface to the atmosphere through conduction and convection (Hu et al. 1999):

$$H = p \cdot c_{\mathrm{p}}\frac{{\left( {T_{\mathrm{s}} - T_{\mathrm{a}}} \right)}}{{r_{\mathrm{a}}}},$$

with *p* as the sea level air density (set to 1.225 kg/m^3^), *c*_p_ as the isobaric mass heat capacity (set to 1.0035 J/kg/K), *r*_a_ as the aerodynamic resistance for an approximated grassland surface (set to a factor of 208 divided by the horizontal wind speed at 2 m height, according to Allen et al. 1998), and *T*_s_ and *T*_a_ as, respectively, the land surface and mean air temperature given in Kelvin. The potential temperature (*θ*) from Eq. 1 describes the temperature of an unsaturated part of dry air when brought adiabatically and reversibly from its initial state and to a standard pressure (Stull 1988):

$$\theta = T_{\mathrm{a}}\left( {\frac{{p_0}}{p}} \right)^{\mathrm{k}},$$

with *T*_a_ is the mean air temperature given in Kelvin, *p*_0_ is the sea level standard air pressure given in millibar, *p* is the atmospheric boundary layer (ABL) air pressure at 1 km a.s.l. (set to 898.7457 millibar), and *k* as the Poisson constant for dry air (set to 0.2854).

#### Estimation of orographic updrafts

The orographic updraft velocity was estimated at 10 × 10 m spatial resolution based on climate variables and a high-resolution DTM downloaded from the Norwegian Geospatial Data Catalog (www.geonorge.no). The orographic updraft velocity (*w*_0_) is a function of horizontal wind speed (*v* in m/s) forced upwards by elevated topography and was estimated according to Brandes and Ombalski (2004) and Bohrer et al. (2012):

$$w_0 = v \cdot C_\alpha.$$

The updraft coefficient (*C*_α_) was calculated as a function of the horizontal wind direction (*α*) in degrees, and the terrain slope (*θ*) and aspect (*β*) angle in degrees calculated from the DTM using the ArcGIS slope and Aspect tools:

$$C_\alpha = {\mathrm{Sin}}\left( \theta \right) \cdot {\mathrm{Cos}}\left( {\alpha - \beta } \right).$$

#### Mapping the seasonal updraft landscape

To compare the seasonal differences in the updraft landscape of Hitra, thermal, and orographic uplift velocities were modeled for one snow-free and cloud-free day for each year from 2013 to 2016 and during the following predefined seasonal periods: January–March (winter), April–June (spring/early summer), July–September (late summer/fall), and October–December (late fall/early winter). As Landsat 8 was launched in February 13th 2013, the study period was restricted to the last 9 months of 2013 (there were no suitable Landsat 8 images for January–March; *n* = 3), 2014 (*n* = 4), 2015 (*n* = 4), and 2016 (until to the end of the study period mid-September 2016, *n* = 3). Given the limited number of available relatively cloud-free images in the different seasonal periods, each image was visually inspected and selected as near mid-season as possible to represent each seasonal period, totaling 14 images.

Landsat 8 Operational Land Imager (OLI) and Thermal Infrared Sensor (TIRS) imagery (Thermal Band 10) were downloaded from the United States Geological Survey archive (United States Geological Survey 2017). The Landsat 8 Quality Assessment Band (BQA) provides pixel-by-pixel information of detected details such as terrain occlusion, water, vegetation, snow, ice, aerosols, clouds, and cloud shadows to mention some. To identify where these characteristics occurred in a given Landsat 8 image, the open source tool “Landsat Land Data Operational Product Evaluation Tool (LDOPE)” (Borak et al. 2002) was used to derive quality issue masks from the BQA-band. Atmospheric correction data (atmospheric transmission constant, upwelling, and downwelling radiance constants; Table 1) for the downloaded Landsat 8 images was calculated with the atmospheric correction parameter calculator (Barsi et al. 2003, 2005). The land surface temperature (*T*_s_) was calculated in ESRI ArcGIS Advanced 10.3 using the Python algorithms for automated mapping of land surface temperature from Landsat 8 images (Walawender et al. 2012). These algorithms are incorporated in the thermal updraft tool, so that it automatically requesting the required atmospheric constants when running the tool. Further details on the exact methodology for calculating LST are presented in Walawender et al. (2012).

**Table 1:** Landsat 8 image acquisition dates, climate variables, and atmospheric correction parameters. No usable Landsat 8 images were available for January-March 2013

Year	Landsat 8 image		Atmospheric transmission constant	Upwelling radiance constant	Downwelling radiance constant	Wind direction (°)	Wind speed (m/s)	Air pressure (millibar)	Mean air temperature (K)
Season	Acquisition date	Quality issues							
2013									
January–March	–	–	–	–	–	–	–	–	–
April–June	May 30th	No	0.81	1.31	2.20	228	8.9	1014.0	299.55
July–September	September 12th	No	0.79	1.46	2.39	42	4.3	1013.6	287.15
October–December	October 21st	Yes	0.93	0.42	0.73	171	8.9	1010.8	278.25
2014									
January–March	March 23rd	Yes	0.94	0.33	0.57	164	7.9	990.8	278.45
April–June	June 2nd	No	0.89	0.74	1.26	62	6.0	1020.4	286.55
July–September	August 30th	No	0.86	1.00	1.68	109	6.9	1012.4	290.55
October–December	October 17th	Yes	0.95	0.33	0.57	124	5.8	1013.7	278.75
2015									
January–March	March 17rd	Yes	0.93	0.43	0.74	70	3.2	1036.4	279.25
April–June	June 21st	No	0.91^*^	0.61^*^	1.03^*^	356	7.2	1013.6	283.55
July–September	August 24th	No	0.9	0.74	1.26	52	10.4	1015.0	294.35
October–December	October 11th	Yes	0.85	0.95	1.59	159	4.1	1028.0	282.35
2016									
January–March	March 28th	No	0.94	0.38	0.65	144	11.1	991.3	281.05
April–June	June 16th	No	0.77	1.52	2.47	10	7.0	1007.0	284.35
July–September	August 19th	No	0.8	1.45	2.39	80	4.6	1011.7	289.75

The Atmospheric Calculation Calculator failed in calculating values for 21st of June 2015 (indicated with *), therefore the correction parameters of 20th of June 2015 was used instead. This was deemed to be acceptable at this aggregated level for the calculation of LST as the weather conditions were comparable for these two dates (16.7 °C and 7.1 m/s for 20th of June 2016 and 13.0 °C and 7.8 m/s for 21th of June 2016)

For each seasonal representative image, climate variables (mean air temperature (*T*_a_), horizontal wind speed (*v*), wind direction (*α*), sea level standard air pressure (*p*_0_)) measured at the Sandstad II weather station at Hitra (63.52° N, 9.11° E; 13 m above sea level) were downloaded from the Norwegian Meteorological Survey (Norwegian Meteorological Survey 2017) for the acquisition date and the required atmospheric correction parameters were calculated with the atmospheric correction parameter calculator as described above (Table 1). All modeling of the updraft landscape of Hitra, given by its thermal and orographic updraft potential, was done in ESRI ArcGIS 10.3 ModelBuilder (model provided as Supplementary Material) using a combination of a high-resolution DTM, remote sensing imagery, climate data, and atmospheric constants.

### Forecasting Risk-Enhancing Terrain for Bird Collisions with Wind Turbines

#### Calculation of minimum sink speed for white-tailed eagles

Different bird species have different wing morphology and mass-to-wing area ratio which affect their flight. These characteristics will influence species-specific flight strategies, and the different bird species’ capability to exploit orographic and thermal uplift as they traverse the landscape. Birds need to overcome drag to enable gliding flight, which The open source Flight program developed by Pennycuick (2008) was used to determine the minimum requirements of white-tailed eagles for taking advantage of uplift in soaring flight activities (i.e., based on the glide polar). Based on the species’ mass (4.8 kg), morphology (wingspan: 2.2 m; wing area: 0.615 m^2^) and air density (1.216 kg/m^3^ at 80 m above sea level), a minimum sink rate of 0.75 m/s for white-tailed eagles was calculated which represents the minimum uplift required to avoid sinking downwards in upward air currents. In ridge soaring conditions, soaring flight is typically used to cover the maximum distance and would therefore be flown at best glide velocity, which is slightly higher than the minimum sink velocity and will result in a slightly higher sink rate (0.93 m/s). The lower minimum sink velocity was used as a threshold to assess the spatial extent of areas with updraft velocities enabling soaring, and to compare to the modeling outcomes (see below).

#### Predicting white-tailed eagle updraft preferences

**Fig. 2:**
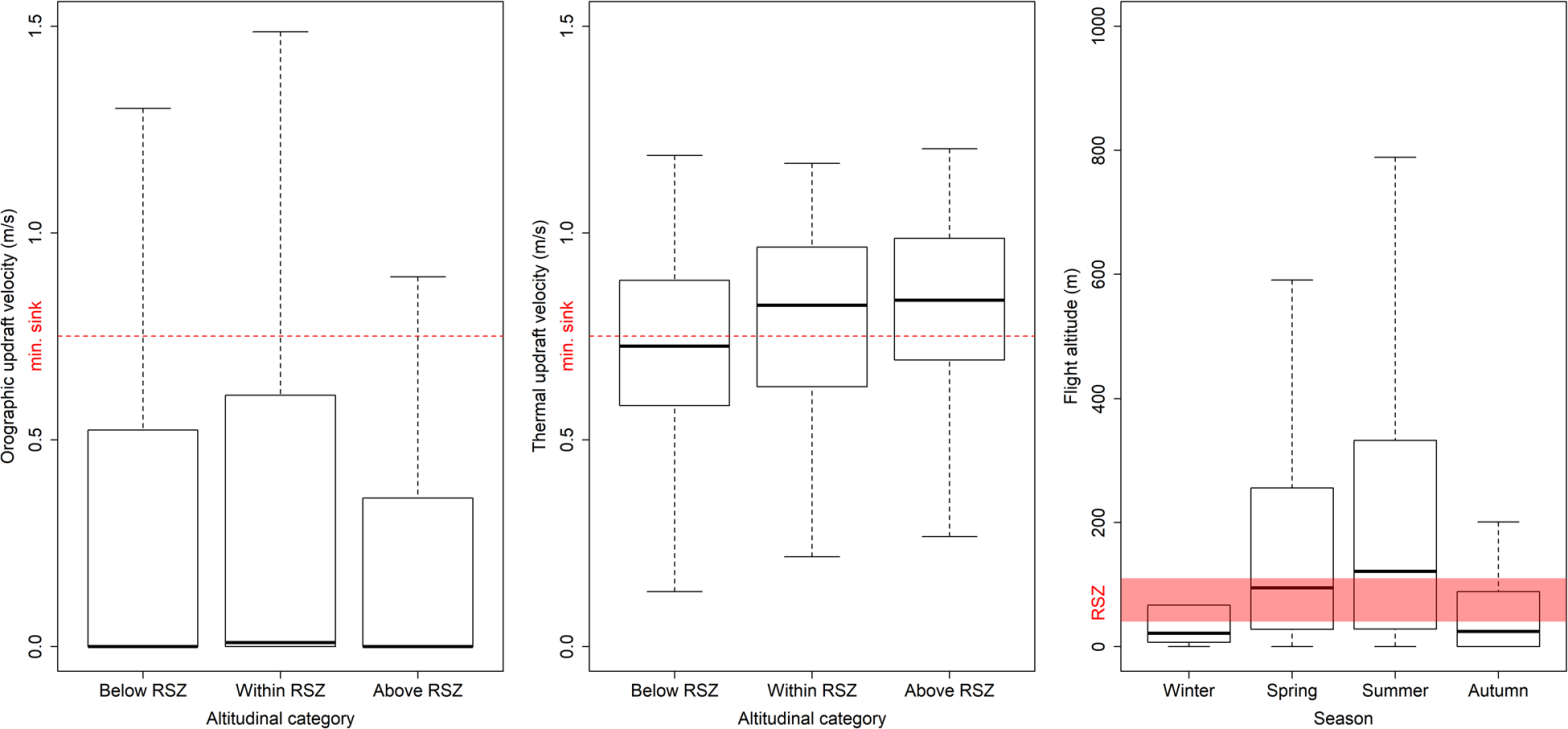
Orographic and thermal updraft associations and seasonal variation in flight altitude on the island of Hitra, for GPS positions of white-tailed eagles. The boxplots show the median (black line), 75 (white box) and 95 (whiskers) percentiles. The two left-most panels include the minimum sink threshold speed for white-tailed eagles (0.75 m/s; red dotted line). The right-most panel includes the altitudinal range falling within the rotor-swept zone (RSZ)

An assessment was performed on whether locations with higher orographic and/or thermal updraft velocities pose increased the potential risk of collisions with wind turbines (when sited at such locations) for white-tailed eagles. Between 2003 and 2016, 71 white-tailed eagle nestlings were equipped on the neighboring island of Smøla with GPS backpack tracking devices (Microwave Telemetry, Inc., Columbia, MD, USA; S.D. ±11 m) rendering data on their movements (Watson et al. 2018). Capture and handling of birds was approved by the Norwegian Environment Agency and the Norwegian Animal Research Authority. The GPS tracking devices were programmed differently per individual with regard to the amount of daily positions acquired (range: 1–24 per day; Nygård et al. 2010). Many of these birds also frequented the island of Hitra. The reason for executing the study at Hitra, was that Smøla has no topography of significance and thereby lacking orographic uplift potential. Because most of the GPS tracking devices were solar-powered, fewer data were obtained during the dark winter months. In the analysis, only in-flight positions within the period 2013–2016 were included with instantaneous speed (i.e., speed when the fix was acquired) larger than 0 m/s and GPS-measured altitude above ground level (a.g.l.) (Poessel et al. 2018). For all included positions, flight altitude was calculated by subtracting the elevation at ground level (DTM 10, ±2–6 m) from the GPS-measured altitude (S.D. ±20 m). Flight altitude was thereafter grouped into three categories: below the rotor-swept zone (RSZ) (<40 m), within RSZ (40–110 m), and above RSZ (>110 m) (Fig. 2).

The probability of presence of an individual in flight was modeled as a binomial variable (1 = GPS positions and 0 = pseudo-absences from random points, respectively) given as a function of the orographic and thermal updraft values. For each position, five random positions (pseudo-absences) were calculated and assigned the same temporal, altitude, and individual information to analyze the differences between actual positions (GPS positions) and available positions (random points generated using the ArcGIS tool “Create random points”). Random positions were distributed throughout the island indicating availability, as white-tailed eagles are known to be able to cover large distances and thus could at any time have traversed the island and surroundings (Nygård et al. 2010). To avoid any spurious results due to potential updraft influences from the surrounding ocean areas, all positions within 100 m of the coastline were removed. The seasonal orographic and thermal updraft values from the 14 season-specific and year-specific datasets were extracted for both the GPS and the random positions assuming the chosen dates (see Table 1) as being representative for all positions within that season and year, to obtain a dataset of used positions with dates and updraft associations and pseudo-absence positions with the same dates and their updraft associations. Only positions with valid updraft values were included, excluding locations with orographic updraft values <0. Such values may occur due to turbulent eddies and lee waves, which—although being important ecological phenomena—are too complex to model in fine detail (Bohrer et al. 2012). Although areas with thermal updrafts have to be balanced with thermal downdrafts elsewhere, these were not accounted for as the methodology calculates the thermal updraft velocity at each location at turbine height and not where this would result in updrafts or downdrafts. Updraft velocities may vary in space and time, and this variability may be affected by topography, time-of-day, season, and wind conditions (Berg et al. 2017). Especially in mountainous terrain, convective boundary layer heights are complex and difficult to model due to the multitude of processes acting simultaneously over a range of spatial and temporal scales (De Wekker and Kossmann 2015). Vertical changes in updraft velocities could therefore not be accounted for in this study.

First, the extent (log-transformed) in which flight altitude of the eagles’ GPS positions was affected by seasonal period, and orographic and thermal updraft velocity was assessed using a linear mixed-effects model while controlling for random effects of season nested within year (1|Year/Season) and hour-of-the-day nested within individuals (1|Ind/Hour) using the lmer function of the lme4 library (Bates et al. 2015).

The probability of presence was thereafter modeled as a function of orographic and thermal updrafts (fixed effects) using generalized linear mixed-effects models with a binomial distribution, while controlling for random effects of seasonal period nested within year (1|Year/Season) and hour-of-the-day nested within individuals (1|Ind/Hour) using the glmer function of the lme4 library (Bates et al. 2015). In total 16 a priori models were compared, assessing the single and additive effects of orographic and/or thermal updraft velocities on the probability of presence, based on the Akaike Information Criterion corrected for small sample sizes (AICc). To assess whether the white-tailed eagles’ response to updrafts was affected by either flight altitude categories or seasonal period, these four basic models (intercept, two single and one additive model) were considered as well as models including first order interactions with flight altitude categories or seasonal periods (Table 2). Model performance of the most parsimonious model (the simplest model with the least assumptions and variables but with greatest explanatory power) was assessed using 10-fold cross-validation employing an adjusted kxvlmer function (Wiens et al. 2008) to evaluate glmer models (kxvglmer). This was done by training the model on a random sample of 90% of the data and testing the goodness-of-fit of the remaining 10% using Pearson correlation within ten randomly assigned frequency bins. To assess the accuracy of the predictive power of the best model influence-curve-based confidence intervals were calculated for cross-validated area under the curve (AUC) estimates using the ci.cvAUC function with ten folds of the cvAUC library (LeDell et al. 2015). Effect sizes for the different covariates are indicated by the *F* statistic. All modeling was scripted (provided as Supplementary Material) in the statistical software program R version 3.2.2 (R Core Team 2015).

**Table 2:** Model parsimony for the a priori models assessing white-tailed eagles’ probability of presence for locations with orographic (O) and/or thermal (T) uplift, also including first-order interactions with flight altitude (F) and/or seasonal period (S)

Model	df	AICc	ΔAICc
O*F+T*F	13	14,321.2	0.0
O*S+T*F	17	14,329.0	7.8
O+T*F	11	14,340.1	18.8
O*F+T*S	17	14,385.7	64.5
O*F+T	11	14,389.5	68.3
O*F	10	14,416.3	95.1
T*F	10	14,417.5	96.3
O*S+T*S	16	14,442.1	120.8
O*S+T	13	14,446.5	125.2
O+T*S	13	14,462.8	141.6
O+T	7	14,464.6	143.4
O*S	12	14,479.3	158.0
O	6	14,493.9	172.6
T	6	14,549.6	228.4
T*S	12	14,549.7	228.5
Intercept	5	14,581.1	259.8

Model parsimony is based on the Akaike Information Criterion corrected for small sample sizes (AICc)

#### Evaluation of potential collision risk at wind turbines

Finally, the maximum orographic and thermal updraft velocities were extracted for the 24 currently installed wind turbines (Hitra I) as well as for the planned sites for the 26 wind turbines still to be constructed (Hitra II) (SAE Vind 2010) to assess potential collision risk at those sites (Fig. 1). Ten white-tailed eagles are known to have collided at turbines in Hitra I (between August 2006 and June 2016), however there have not been executed intensive searches for collision victims. This assessment should therefore solely be considered as a mapping of potential risk at appropriate turbine sites.

## Results

Seasonal orographic and thermal updraft velocity maps were estimated for the entire island for the selected dates in Table 1. The estimated thermal updraft velocities ranged from 0 to 1.28 m/s and the estimated orographic updraft velocities ranged from 0 to 9.89 m/s. White-tailed eagles require an uplift velocity above 0.75 m/s (minimum sink rate) to take advantage of the updrafts in their soaring flight activities. When using this threshold value to segment the maximum updraft maps across seasons, potential thermal soaring areas as well as potential ridge-lift areas available for white-tailed eagles can be identified (Fig. 1). The spatial distribution of thermal uplift areas was significantly more spatially homogenous than the small and fragmented orographic uplift areas. The orographic uplift patches along hills and ridges provide pockets of stronger orographic updraft velocities compared to the relatively weaker updraft velocities in the thermal uplift areas. 93.6% (637 km^2^) of the total land area of Hitra provide thermal updraft velocities >0.75 m/s, whereas only 53.9% (366.6 km^2^) provide orographic updraft velocities >0.75 m/s.

Altogether 16 birds were represented in the dataset (2759 GPS positions and 12,882 random positions). Because the GPS devices were solar-powered, the number of GPS positions varied by season (winter <1%, spring 42%, summer 54%, and autumn 4%). In flight, the eagles spent 31% of their time below RSZ, 20 % within RSZ and 49% above RSZ. Flight altitude (log-transformed) of GPS positions varied significantly by seasonal period (*F* = 5.68, *P* < 0.001; Fig. 2) with higher flight altitude during spring compared to winter. However, this result should be interpreted with caution. Flight altitude of GPS positions increased with thermal updraft velocities, but not orographic updrafts (respectively: *F* = 27.45, *P* < 0.001 and *F* = 0.46, *P* = 0.496; Fig. 2).

**Fig. 3:**
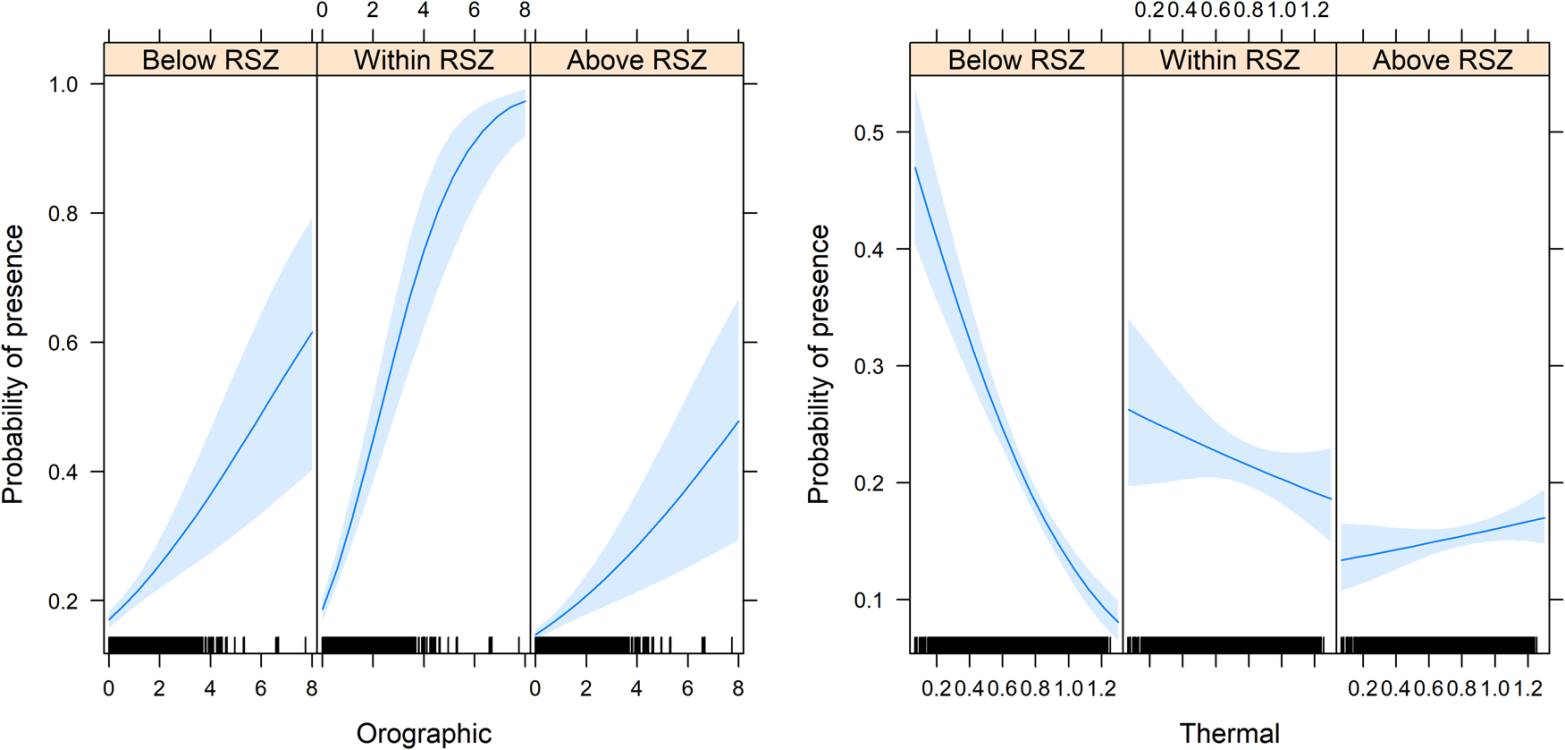
Partial effects of orographic (left) and thermal (right) updrafts, respectively, in interaction with flight altitude category (below, within and above the rotor-swept zone (RSZ)) on the probability of presence in white-tailed eagles on the island of Hitra. The shaded areas indicate the 95% confidence interval

The additive effects of orographic and thermal updraft velocities in interaction with flight altitude rendered the most parsimonious model in explaining selection probability (Table 2, AICc = 14,321.2). The probability of presence of flying white-tailed eagles in this model (cross-validation: $\bar r$ = 0.855, *P* = 0.004; AUC 0.578 ± 0.006 S.E.) increased significantly with orographic updraft velocity (*z* = 4.459). White-tailed eagles’ response to orographic uplift was strongest at flight altitudes within range of the rotor-swept zone (RSZ: 40–110 m a.g.l.; *z* = 3.920) (Fig. 3). Whereas, the probability of presence was negatively correlated with thermal updraft velocity below RSZ (*z* = −9.782), it increased with higher flight altitudes (*z* = 4.823 and *z* = 8.385 for within and above RSZ, respectively; Fig. 3). Flight altitude category had a significantly stronger effect on probability of presence than seasonal period, given that the highest-ranking models all included flight altitude category (Table 2).

**Fig. 4:**
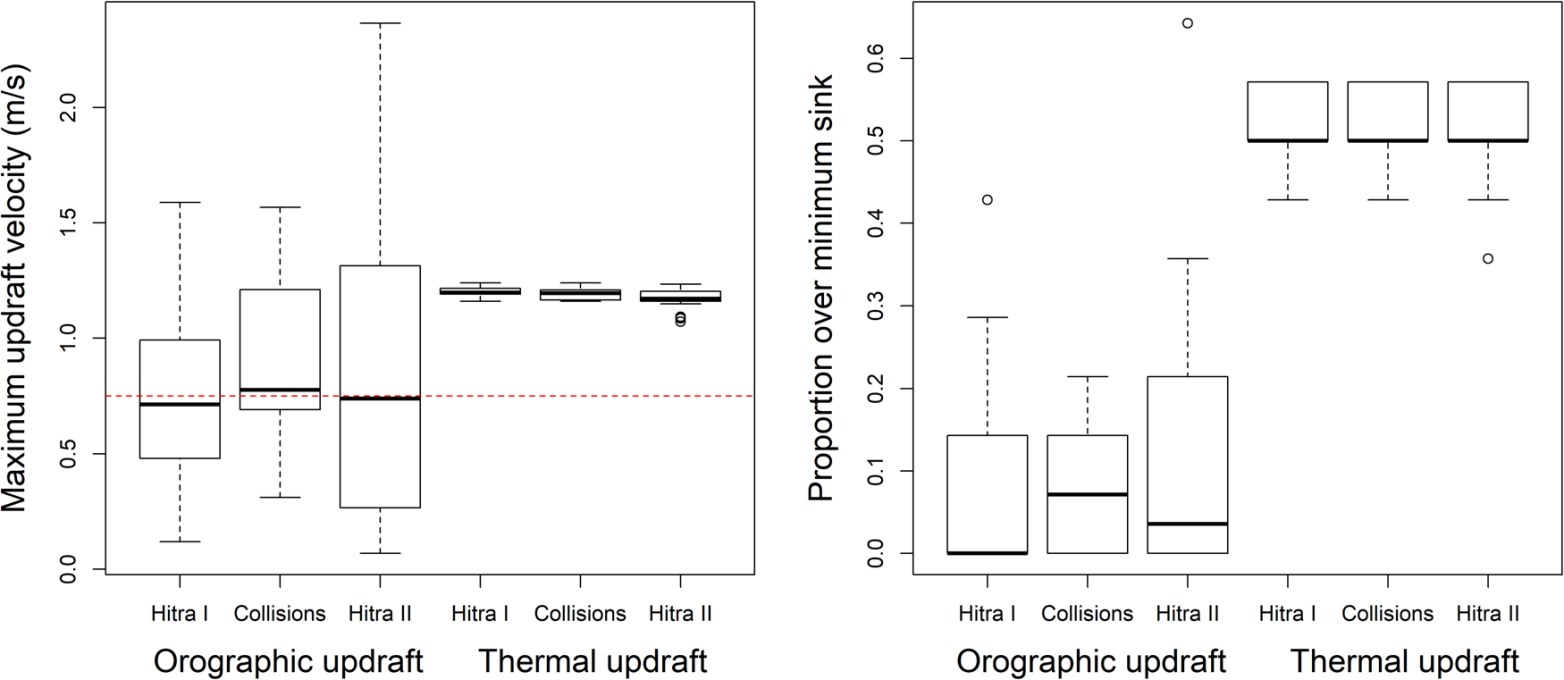
Updraft velocity (m/s) and proportion of seasons (Spring 2013–Summer 2016, *N* = 14) exceeding minimum sink rate for white-tailed eagles (0.75 m/s; red dotted line) regarding orographic and thermal updrafts at 24 constructed (Hitra I) and 26 planned (Hitra II) wind-turbine locations as well as recorded bird collisions on the island of Hitra, Norway. The boxplots show the median (black line), 75 (white box), and 95 (whiskers) percentiles

The wind turbines within the Hitra wind-power plant were placed at locations rendering maximum orographic updraft velocities around the minimum sink rate for white-tailed eagles (medians for Hitra I and II: 0.72 and 0.74 m/s) but higher maximum thermal updraft velocities (medians for Hitra I and II, respectively: 1.20 and 1.17 m/s) (Fig. 4). While the turbine sites barely obtained increased orographic updrafts across seasons, thermal updrafts exceeded the minimum sink rate of 0.75 m/s in half of the seasons. Turbines where collisions were recorded rendered similar results (medians for orographic and thermal updraft velocities, respectively: 0.78 and 1.20 m/s). The orographic updraft velocities were slightly over the minimum sink speed, however, this was not significantly different compared to non-collision turbines (Mann–Whitney *U*-test: *W* = 50, *P* = 0.576).

## Discussion

In this study, the uplift landscape of Hitra was estimated and spatially visualized based on a high-resolution DTM and Landsat 8 Thermal Band 10 imagery. This high-resolution modeling approach refers specifically to the spatial resolution, not the temporal resolution. As the modeling depends on good quality remote imagery (e.g., no ice, snow, and clouds) (Li et al. 2013; Tomlinson et al. 2011) at a fine spatial resolution it does not allow for a fine temporal resolution, capturing within-day variance in wind speed or radiation. However, for micrositing of wind turbines, the spatial resolution will be of highest importance. As illustrated in Fig. 1, these relatively small and fragmented orographic uplift patches along hills and ridges provide pockets with stronger orographic updraft velocities compared to the relatively weaker updraft velocities in the thermal uplift areas. The strongest orographic uplift is associated with strongly negative thermal uplift. This is because strong winds, which are required for orographic uplift, produce shear that tends to break apart thermal formation (Bohrer et al. 2012). The development, strength, and duration of thermal convection is affected by topography as well as changing atmospheric and environmental conditions (Shamoun-Baranes et al. 2003). While the highest levels of thermal uplift were widely found in flat terrain but temporally limited to calm and sunny days, the more commonly occurring orographic uplift was spatially limited to steep slopes and ridges. This clearly shows the importance for high-resolution modeling, as small pockets of strong orographic updrafts may not have been identified at coarser spatial resolutions. It should however be noted that this methodology should mainly be utilized as a spatial proxy to identify wind turbine sites with enhanced collision risk due to uplift conditions as it does not incorporates all environmental and meteorological variability of complex updraft landscapes (Bohrer et al. 2012; Dodge et al. 2014; Santos et al. 2017; Sapir et al. 2011).

From a methodological perspective, the importance of having temporally comparable and coincident climate parameters has to be emphasized, atmospheric correction parameters, and cloud-free images. For the seasonal study periods a total of 14 cloud-free Landsat 8 images were of acceptable quality. Although the small number of Landsat images considered limit the inferences for validation, for thermal updraft estimation at Hitra this was the only available cloud-free images at the spatial resolution of 100 × 100 m of the Landsat 8 Thermal Band 10. Alternative temporal climate data, including interpolated raster grids on air temperature, wind speed, and wind direction from the Norwegian Meteorological Institute, are not meaningful for the rugged terrain of Hitra given their low spatial resolution (1 km^2^). As the Nordic hemisphere average solar declination angle from June to September is 15° N (Stull 1988), thermal updrafts have their optimum in northern mid-latitudes (from 31° to 59° N) during spring into late summer (Bradbury 2000). It is therefore reasonable to expect relatively low thermal updraft velocities at the high latitude of Hitra (63.60° N). In an identical study conducted in the Tarifa region at the Spanish side of the Gibraltar strait (36.0132° N, 5.6027° W), Santos et al. (2017) estimated thermal updraft values for the same season about three times higher than the estimated maximum value for Hitra. Given that wind turbines once sited will operate at that specific location throughout the operational lifetime of the wind-power plant, maximum updraft velocities form a good proxy potential risk of collision from a precautionary perspective. Even though this risk may not necessarily exceed the minimum sink rate for species of concern at all times, those situations will inevitably occur at such sites over time.

Soaring birds are known to use thermal and orographic uplift to gain altitude to save energy (Barrios and Rodriguez 2004; Harel et al. 2016a; Shamoun-Baranes et al. 2003, 2016). The updrafts generated at the topographic locations of wind turbines together with air diverted around the turbines may attract soaring bird species, enhancing collision risk (Barrios and Rodriguez 2004; de Lucas et al. 2012; Drewitt and Langston 2008; Krijgsveld et al. 2011). The validation of this approach at Hitra, as well as a similar study executed in Tarifa (Gibraltar, Spain) (Santos et al. 2017), indicated a significant correlation between the fine-scale distribution of especially orographic updrafts and raptor flight activity. White-tailed eagles preferred to utilize the relatively fragmented patches with strong orographic updraft velocities. Their probability of presence at locations with uplift depended on their flight altitude. Their response to orographic uplift was strongest at low flight altitudes; generally, within the range of the rotor swept zone. Contrary to this, the white-tailed eagles were not selecting the more widely distributed areas with weaker thermal updraft velocities. Katzner et al. (2012) found that golden eagles *Aquila chrysaetos* flew at lower altitudes over steep slopes and cliffs (where orographic lift can develop) compared to flights over flats and gentle slopes (where thermal lift is more likely). Santos et al. (2017) found that black kites *Milvus migrans* flew at lower altitudes above ground level during linear soaring compared to circling. This study also confirmed that white-tailed eagles flew higher at locations with higher thermal updraft velocities. Although the negative association with thermal uplift to the probability of presence may seem counterintuitive, the thermal updraft velocities never reached as high values as did orographic updraft. Most studies on soaring behavior fueled by uplift have been carried out at southern latitudes, where the thermal uplift component is much stronger than in Norway (Barrios and Rodriguez 2004; Harel et al. 2016a; Shamoun-Baranes et al. 2003, 2016; Treep et al. 2016). White-tailed eagles can therefore not be expected to actively seek out such areas at high latitudes. Even though white-tailed eagles are known to use circling flight at higher flight altitudes on calm, warm, and sunny days (pers. obs.), such days do not occur often at the northern latitudes of Hitra. Although such circumstances may allow for staying aloft over long periods with minimum expenditure of effort (Pennycuick), it will not be a reliable way for cross-country soaring. Linear soaring along steep slopes and ridges will provide white-tailed eagles with a much more efficient cross-country flight strategy during the more usual windy conditions. Ridge soaring can be operated at best glide speed, a more efficient condition than minimum sink speed. White-tailed eagles will unlikely be able to benefit from both types of uplift conditions given the strong negative correlation between areas (slopes versus flat terrain) and periods (windy and cloudy versus calm and sunny), where and when orographic versus thermal updrafts are strongest (cf. Santos et al. 2017). Still, to enable identifying temporal thermal “hotspots”, choosing Landsat images on such days is warranted with regard to micrositing of wind turbines. The local patches of strong orographic updrafts providing low-flying soaring birds with the required uplift above the minimum sink speed will therefore be most important with regard to risk for collision.

The choice of placement of the wind turbines within the landscape can thus minimize the exposure of birds to the hazard posed by those wind turbines (May et al. 2015). This study indicated that the current and proposed turbines on the island of Hitra were sited at locations with maximum orographic updraft velocities around the minimum sink rate for white-tailed eagles. Still, the turbines were sited at locations with higher maximum thermal updraft velocities potentially leading to temporal exposure to increased collision risk on warm and sunny days. Developers typically seek to locate turbines, where wind conditions and inter-turbine spacing are optimized with respect to wind capture and minimized wake effects (Herbert-Acero et al. 2014; Serrano González et al. 2014). Wind turbines are often sited on hilltops or along ridges where they are well-exposed to prevailing winds from all directions, and where wind speeds are higher due to upward compression (caused by the Venturi or Bernoulli effect) on the windward side of the topography (Whiteman 2000). These upward, orographic, air currents are the same which are utilized by soaring birds to gain altitude (Katzner et al. 2012; Miller et al. 2014). This causes a trade-off situation for micrositing of wind turbines between bird-attractive updraft locations and locations attractive for wind yield. However, having access to (proxy) information on both—potentially conflicting—aspects during the design phase, enables developers to make a trade-off decision minimizing the potential collision risk per kWh when micrositing wind turbines (Bohrer et al. 2013; Liechti et al. 2013). Micrositing has so far been proposed in agricultural areas (Mammen et al. 2011) and wetlands (Hill et al. 2011), and especially along ridges with many soaring and migratory raptors (Barrios and Rodriguez 2004; de Lucas et al. 2012; Katzner et al. 2012; Kitano and Shiraki 2013; Smallwood and Thelander 2008). Although proposed by several, it remains unclear whether micrositing practice has resulted in adjusted design of wind-power plants. This is probably because it is mainly promoted as part of the project-by-project consenting process and mitigation requirements, and not (yet) integrated into standard wind project design and optimization software (e.g., Openwind, WindFarmer, and WindPro) such as noise, visibility, and shadow flickering (Herbert-Acero et al. 2014; Serrano González et al. 2014). Forecasting landscape features that enhance potential risk for soaring raptors could, however, be implemented cost-effectively in the pre-construction assessment for improved micrositing of wind turbines to reduce environmental impacts and associated economic risk.

## Conclusion

This study presents a cost-effective high-resolution orographic and thermal updraft modeling tool, based on a combination of GIS and remote sensing imagery, for the identification of risk-enhancing landscape features that can be used to predict the probability of presence of soaring raptors. The validation of this tool, performed at Hitra and in Tarifa (Gibraltar, Spain) (Santos et al. 2017), indicated a significant correlation between the fine-scale distribution of especially orographic updrafts and GPS-tracked raptor flight activity. The developed tool is easy to implement anywhere using publicly available high-resolution digital terrain models and cloud-free satellite imagery. Moreover, the method is flexible with respect to the local availability of climate parameters and atmospheric correction parameters as well as the bird species considered. This study may contribute to improved preconstruction assessment of wind-power plants through “bird-friendly” micrositing of wind turbines, and hence reduce the environmental impacts for soaring raptors.

## Data Availability

All Landsat 8 imagery for the validation are available from USGS Earth Explorer (https://earthexplorer.usgs.gov/). The DTM10 for the validation is supplied as supplementary materials at NINAs geodata portal (https://geodata.nina.no/layers/geonode:dem10utm). All image acquisition dates, and atmospheric and climatic constants are listed in Table 1 in the submitted manuscript.

## Code availability

Codes for the updraft calculations are supplied as supplementary materials at NINAs GitHUB repository (https://github.com/NINAnor/INTACT_Micrositing). The R scripts and data files to perform the white-tailed eagle validation are supplied as supplementary materials at the Mendeley Data repository (10.17632/pkbsnftv5h.1).
